# Disrupted Functional Network Connectivity Predicts Cognitive Impairment in Presbycusis Patients

**DOI:** 10.3389/fnagi.2020.00246

**Published:** 2020-08-12

**Authors:** Chunhua Xing, Juan Zhang, Jinluan Cui, Wei Yong, Jinghua Hu, Xindao Yin, Yuanqing Wu, Yu-Chen Chen

**Affiliations:** ^1^Department of Radiology, Nanjing First Hospital, Nanjing Medical University, Nanjing, China; ^2^Department of Neurology, Nanjing Yuhua Hospital, Yuhua Branch of Nanjing First Hospital, Nanjing, China; ^3^Department of Otolaryngology, Nanjing First Hospital, Nanjing Medical University, Nanjing, China

**Keywords:** presbycusis, cognitive impairment, brain network, resting-state fMRI, functional network connectivity

## Abstract

**Purpose**: Individuals with presbycusis often show deficits in cognitive function, however, the exact neurophysiological mechanisms are not well understood. This study explored the alterations in intra- and inter-network functional connectivity (FC) of multiple networks in presbycusis patients, and further correlated FC with cognitive assessment scores to assess their ability to predict cognitive impairment.

**Methods**: Resting-state functional magnetic resonance imaging (rs-fMRI) was performed in 40 presbycusis patients and 40 matched controls, and 12 resting-state networks (RSNs) were identified by independent component analysis (ICA) approach. A two-sample *t*-test was carried out to detect the intra-network FC differences, and functional network connectivity (FNC) was calculated to compare the inter-network FC differences. Pearson or Spearman correlation analysis was subsequently used to explore the correlation between altered FC and cognitive assessment scores.

**Results**: Our study demonstrated that patients with presbycusis showed significantly decreased FC in the subcortical limbic network (scLN), default mode network (DMN), executive control network (ECN), and attention network (AN) compared with the control group. Moreover, the connectivity for scLN-AUN (auditory network) and VN (visual network)-DMN were found significantly increased while AN-DMN was found significantly decreased in presbycusis patients. Ultimately, this study revealed the intra- and inter-network alterations associated with some cognitive assessment scores.

**Conclusion**: This study observed intra- and inter-network FC alterations in presbycusis patients, and investigated that presbycusis can lead to abnormal connectivity of RSNs and plasticity compensation mechanism, which may be the basis of cognitive impairment, suggesting that FNC can be used to predict potential cognitive impairment in their early stage.

## Introduction

Presbycusis, also known as age-related hearing loss, results from lifetime damage to the auditory system and can be defined as progressive bilateral sensorineural high-frequency hearing loss (Gates and Mills, 2005). Presbycusis has become the third leading chronic health disorder affecting elderly over the age of 65 after hypertension and arthritis (GBD 2015 Disease and Injury Incidence and Prevalence Collaborators, 2016), which mainly characterized by slow central processing of acoustic information, impaired localization of sound sources, and reduced ability to distinguish speech under noisy environments (Gates and Mills, 2005; Tavanai and Mohammadkhani, 2017). Lots of robust research evidence suggests that presbycusis not only causes alterations of function and morphology in specific brain areas, but also independently associated with cognitive decline, increasing the risk of dementia, and impaired performance across cognitive domains (Thomson et al., 2017; Ford et al., 2018; Loughrey et al., 2018), this results in a serious adverse impact on their daily life and social interaction of elderly. Early detection and prevention have supposed to be the most potential approaches in dealing with cognitive impairment. Therefore, the recognition of early cognitive impairment is of great importance for patients with presbycusis.

Resting-state functional magnetic resonance imaging (rs-fMRI) does not require subjects to receive any sensory stimuli or perform a specific task, thus, it is widely used in describing abnormal brain neuronal activity and functional connectivity (FC) in various clinical conditions (Biswal et al., 1995). FC refers to measuring the temporal synchronization of neuronal activity between different brain regions within resting-state networks (RSNs), reflecting the functional status of the corresponding brain regions (Geerligs et al., 2015). Therefore, the use of this technology to map the Spatio-temporal covariance structure of spontaneous brain activity networks is increasing, which will provide an in-depth understanding of the neural mechanisms for cognitive impairment in presbycusis. A growing body of research has indicated that auditory and cognitive processing are tightly related and that patients with hearing loss recruit functions of the executive network to maintain communication, leading to cascading cognitive effects that further affect comprehension, perception and working memory (Peelle and Wingfield, 2016; Loughrey et al., 2018). Previous research has found that hearing loss was associated with decreased volume in the temporal lobe, which is responsible for semantic memory and sensory integration, and the atrophy of the temporal lobe may be involved in the early stage of mild cognitive impairment (Fortunato et al., 2016). Another finding suggested that during effortful speech perception in the hearing-impaired showed increased activation of the frontal lobe, which leads to fewer resources of frontal lobes for cognition and indirectly affected the high-level cognitive processes. Moreover, hearing loss can lead to impaired auditory-limbic network FC (Rutherford et al., 2018), and resulted in a cross-modal plastic reorganization of the auditory cortex. Even mild hearing loss affects the transmission of information within the auditory-linguistic-motor circuits (Bidelman et al., 2019).

Taken together, cognitive impairment in presbycusis most likely depends on system-level disruption of brain networks, namely the internal interactions of different brain regions in one network or the interactions among multiple networks, rather than the dysfunction of a single discrete brain region. However, conventional seed-based FC researches rely on the user’s self-defined region of interest (ROI; Lv et al., 2018) and fail to fully investigate the interaction between the brain networks of presbycusis patients. Independent component analysis (ICA) using a data-driven method (McKeown et al., 1998) without prior experimental models or assumptions decompose BOLD signal from the whole brain voxels into spatially and temporally independent components (ICs), which has been widely used in rs-fMRI and is capable of measuring interactions within and between multiple brain networks directly. Among them, resting-state function network connectivity (FNC) can be used to describe the temporal correlation between these RSNs (Wang et al., 2014; Qin et al., 2018). At present, ICA studies on the connectivity changes within and between networks in presbycusis have not been reported, just a few studies focused on intra-network FC alteration (Schmidt et al., 2013; Luan et al., 2019). Therefore, we predict that by exploring RSNs and FNC to elucidate the impairment and compensation patterns of cognitive impairment in presbycusis patients will provide valuable information for rational treatment.

This study aims to systematically explore FC and their interactions within and between RSNs in presbycusis patients and to provide new reliable markers for identifying early cognitive impairments. We will experimentally validate the following hypothesis: first, the cognitive impairment in presbycusis patients is related to the disrupted FC of multiple RSNs; second, in addition to changes within the network, changes between networks may also be associated with cognitive impairment.

## Materials and Methods

### Subjects

A total of 40 presbycusis patients were recruited from the otolaryngology department, and 40 age-, gender-, education-, and handedness matched control subjects were selected from community health census or online advertising. Based on the definition of hearing loss, all participants underwent the hearing loss assessment using the speech-frequency pure tone average (PTA) of the 0.25, 0.5, 1, 2, 4, 8 kHz (air conduction) threshold in the better hearing ears. The PTA value of 25 dB HL is the normal listening threshold limit. No significant difference in the auditory threshold between the experimental and control group was observed; and middle ear function was measured by using Madsen Electronics Zodiac 901 Middle Ear Analyzer (GN Otometrics).

Exclusion criteria were as follows: (1) in addition to presbycusis, ear diseases that impacted hearing threshold, including tinnitus, hyperacusis and Meniere’s disease (Lopez-Escamez et al., 2015); (2) a history of otologic surgery, ototoxic drug therapy, noise exposure or hearing aid use; (3) asymmetric hearing loss, with the difference of air conduction threshold exceeding 20 dB, and at least two frequencies between 0.5, 1, 2 and 4 kHz; (4) severe smoking, alcohol abuse, brain damage, Alzheimer’s disease, Parkinson’s disease, major depression, epilepsy, mental or neurological disorders, major diseases (such as anemia, thyroid dysfunction, cancer); and (5) a contraindication to MRI.

### Neuropsychological Assessment

The neuropsychological assessment of all participants required a comprehensive test of cognitive status, including the use of Mini-Mental State Exam (MMSE; Ardila et al., 2016) and Montreal Cognitive Assessment (MoCA; Lu et al., 2011) to assess general cognitive function, Auditory Verbal Learning Test (AVLT and AVLT-delay; Xu et al., 2020) and Complex Figure Test (CFT and CFT-delay; Shin et al., 2006) for episodic verbal learning as well as visual memory recall, Digit Span Test (DST; Gabel et al., 2019) for verbal working memory. Executive control was assessed by Trail-Making Test A and B (TMT-A and TMT-B; Sánchez-Cubillo et al., 2009) and Clock-Drawing Test (CDT; Viscogliosi et al., 2017), besides mental processing speed and visuospatial abilities were evaluated by Digit Symbol Substitution Test (DSST; Rosano et al., 2013) and Verbal Fluency Test (VFT; Brucki and Rocha, 2004). Also, the Self-Rating Anxiety Scale (SAS; Song et al., 2014) and Self-Rating Depression Scale (SDS; Zung, 1971) were used to assess the symptoms of anxiety and depression. There are a total of 14 cognitive tests and it took almost 60 min for each individual to finish this battery of orderly tests.

### Imaging Data Acquisition

All imaging data acquisitions were performed on a 3.0 Tesla Philips MRI scanner (Ingenia, Netherlands) with an eight-channel phased-array head coil. Resting-state functional images were acquired axially using a gradient echo-planar imaging sequence as following parameters: repetition time (TR) = 2,000 ms, echo time (TE) = 30 ms, slices = 36, thickness = 4 mm, gap = 0 mm, field of view (FOV) = 240 mm × 240 mm, acquisition matrix = 64 × 64, and flip angle (FA) = 90°, the voxel size was 3.75 × 3.75 × 4.0 mm^3^; and this sequence lasted 8 min and 8 s. Structural images were obtained using a three-dimensional turbo fast echo (3D-TFE) T1WI sequence and following scan parameters: TR/TE = 8.1/3.7 ms, slices = 170, thickness = 1 mm, gap = 0 mm, FOV = 256 mm × 256 mm, acquisition matrix = 256 × 256, and FA = 8°. The structural sequence lasted 5 min and 29 s. Besides, all scans were acquired with parallel imaging using sensitivity encoding (SENSE) technique and SENSE factor = 2.

During the scan, the participants were instructed to lie quietly and keep still, with eyes closed but not asleep or think about anything special. Meanwhile, foam padding was used to reduce the involuntary movement of the head, and earplugs were used to reduce the influence of noise on the participants. According to the manufacturer’s specifications, the earplugs (Hearos Ultimate Softness Series, USA) could attenuate scanner noise by almost 32 dB.

### Preprocessing of Functional Imaging Data

GRETNA (Graph Theoretical Network Analysis) was applied to preprocess the functional image data for further analysis (Wang et al., 2015), involving the following steps. First, the first 10 volumes were removed to allow for an equilibrium of the magnetization and adaptation of the participants to the scanning environment. Then the remaining volumes were sliced for timing (slice timing) and corrected for head motion (realignment). Since micromovements from volume to volume can influence the FC, framewise displacement (FD) values were computed for each subject to reflect the temporal derivative of the movement parameters. Time points that exceeded a max FD of 0.5 mm were excluded from subsequent analyses. The corrected volumes were spatially normalized to the Montreal Neurological Institute space with resampled voxel size = 3 × 3 × 3 mm^3^, and finally spatially smoothed with a Gaussian smooth kernel (full width at half-maximum of 6 mm).

### Identification of Resting-State Networks

The group ICA software (Medical Image Analysis Lab, University of New Mexico, Albuquerque, NM, USA^1^) was used to implement the spatial group ICA and identify RSNs. ICA analysis is performed in three phases: (1) data reduction; (2) application of ICA algorithm; and (3) back reconstruction for each subject. The number of ICs over all subjects was estimated using the minimum description-length (MDL) criteria. In phase one, principal component analysis (PCA) was used to reduce computational complexity, then the remaining reduction step was achieved again using PCA based on a selected number of ICs. In phase two, the infomax algorithm was used to run the proper ICA. In the final phase, Single-subject individual time courses and spatial maps were group ICA (GICA) type back-reconstructed and results were converted into z-scores to display.

### Intra-network Functional Connectivity Analysis

Among the 40 components arising from ICA, 12 components (eight meaningful RSNs) were selected as the focus of subsequent analysis through visual inspection based on previous rs-fMRI studies. For each RSNs, a one-sample *t*-test was first performed to obtain z-maps for each group, which were corrected by false discovery rate (FDR) method, and the statistical figure was obtained at the threshold of *p* < 0.05. The spatial maps of the components were used as variables for a one-sample *t*-test. Each mask of the control group and presbycusis was further combined into a total mask for each component. Then, the z-maps of RSN were compared between groups using a two-sample *t*-test of voxel restricted within the combination mask. The comparison results were corrected *p* < 0.01, with a Monte Carlo simulation for multiple comparisons (AlphaSim correction^2^), and the regions of significant differences were selected from each RSNs to facilitate further analysis.

### Inter-network Functional Connectivity Analysis

After ICA, the spatiotemporal double regression method was used to obtain the individual level time courses of the identified RSNs. Then, FNC analysis was carried out to study the relationship between different RSNs time courses. During the analysis, a temporal band-pass filter (range from 0.00 to 0.25 Hz) is first applied to the time courses to reduce the effects of low-frequency drift and high-frequency physiological noise. Secondly, the correlations were calculated between any two RSNs time courses of each subject. Then the FNC, also known as temporal correlation, is obtained by calculating the Pearson correlation coefficient of the time courses of selected RSNs and generate the 13 × 13 matrix. A general linear model (GLM) with age, sex as covariates was finally used to analyze which pairs of RSNs were significantly different between controls and patients. The significance threshold was *p* < 0.05, corrected for multiple comparisons using FDR.

### Correlations With Neuropsychological Assessment

To further investigate the relationship between connectivity anomalies and cognitive impairment in patients with presbycusis, we performed the correlation analysis between the abnormal connectivity regions detected and neuropsychological assessment scores. That is, for the inter-network FC, the brain region with a significant difference in the two-sample *t*-test was selected as the ROIs and the coordinates of ROIs were extracted. Then, the mean z-scores within ROI were used to illustrate the correlation. Also, for intra-network FC, significant differences among the three groups were detected at the level of 12 components, and its FNC coefficients were used to calculate the correlation with assessment scores.

### Statistical Analyses

To investigate the difference between presbycusis patients and healthy controls for demographic and clinical information, the chi-square test was applied to the categorical variables and independent two-sample *t*-test for continuous variables, both conducted by the IBM SPSS 19.0 package, *p* < 0.05 was considered statistically significant. Then, we used Cohen’s *d* to describe the effect size (ES) of each clinical variable. Meanwhile, for RSNs and FNC analysis, a two-sample *t*-test was performed to conducted group comparison between presbycusis patients and healthy controls, and results were corrected for AlphaSim (*p* < 0.01) and FDR method (*p* < 0.05), separately. Pearson or Spearman correlation was used to exam the relationship between FC and neuropsychological test scores with a statistical significance level *p* < 0.05. During all this analysis, SPM8 (statistical parametric mapping) was used to carry out the voxel-level statistical analysis of RSNs, and MATLAB function (MATLAB 2013a) was used for FNC group comparison and other correlation analysis. Bonferroni correction for multiple comparisons was used in the correlation analysis.

## Results

### Clinical Characteristics and Neuropsychological Data

**Figure 1:**
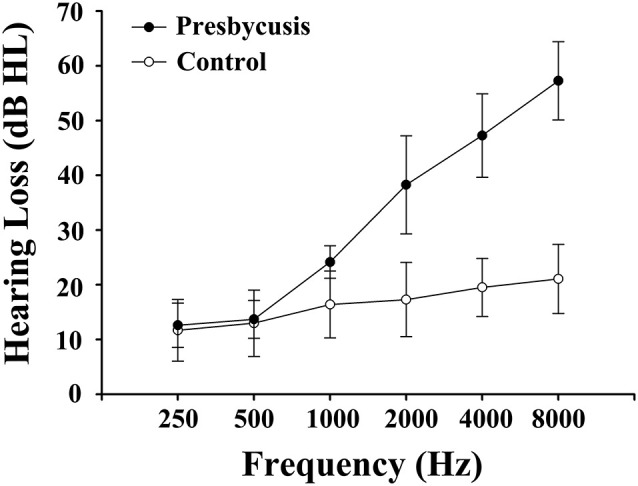
Mean hearing thresholds of the presbycusis and control groups from 250 Hz to 8,000 Hz. Data are presented as mean ± SD.

The clinical characteristics as well as the neuropsychological results of presbycusis patients and the control group were summarized in Tables s 1, 2. There were no significant differences in the aspect of age, gender, and education level. All participants had a type-A tympanometry curve, suggesting the normal function of the middle ear. No significant difference was observed in PTA between the left and right ear of the presbycusis and the control group. The average hearing thresholds of both ears in presbycusis and control group were shown in Figure 1. The average PTA of presbycusis patients was significantly higher than that of the control group (*p* < 0.001, 1,000–8,000 Hz). In neuropsychological assessment, patients with presbycusis performed significantly worse on DST and TMT-B scores (*p* < 0.05). The other neuropsychological tests did not show any significant differences between the two groups. No subjects had max FD > 0.5 mm on more than 35 volumes in this study. No significant difference was found in the mean and max FD values between presbycusis patients and controls.

**Table 1:** Demographics of the presbycusis patients and healthy controls.

	Presbycusis patients (*n* = 40)	Healthy controls (*n* = 40)	*p*-value
Age (years)	61.35 ± 5.05	61.30 ± 3.89	0.961
Sex (male: female)	20/20	17/23	0.501
Education levels (years)	10.45 ± 1.84	10.58 ± 1.53	0.742
PTA of the left ear (dB HL)	32.27 ± 4.71	16.17 ± 2.98	<0.001*
PTA of the right ear (dB HL)	32.88 ± 6.58	16.02 ± 3.17	<0.001*
Mean PTA of both ears (dB HL)	32.32 ± 4.24	15.92 ± 2.58	<0.001*
Mean FD value (mm)	0.20 ± 0.06	0.19 ± 0.06	0.291
Max FD value (mm)	0.44 ± 0.05	0.42 ± 0.08	0.146

Data are represented as Mean ± SD. *P < 0.001. PTA, pure-tone audiometry; FD, framewise displacement.

**Table 2:** Neuropsychological tests of presbycusis patients and healthy controls.

	Presbycusis patients (*n* = 40)	Healthy controls (*n* = 40)	*p*-value	ES
MMSE	29.18 ± 1.06	28.68 ± 1.44	0.081	0.40
MoCA	25.68 ± 1.69	25.93 ± 1.79	0.522	0.14
AVLT	35.35 ± 7.38	34.88 ± 7.75	0.779	0.06
AVLT-delay	7.18 ± 2.23	6.75 ± 2.31	0.405	0.19
CFT	34.70 ± 1.57	34.58 ± 1.56	0.722	0.08
CFT-delay	16.70 ± 3.07	17.20 ± 2.78	0.447	0.17
TMT-A	70.73 ± 21.56	67.60 ± 18.36	0.487	0.16
TMT-B	195.73 ± 63.59	156.03 ± 53.52	0.003*	0.68
CDT	3.55 ± 0.55	3.63 ± 0.54	0.541	0.15
DST	10.65 ± 1.48	11.68 ± 2.08	0.013*	0.57
VFT	13.95 ± 4.05	14.41 ± 3.71	0.596	0.12
DSST	70.13 ± 8.22	68.73 ± 9.94	0.494	0.15
SAS	36.83 ± 5.82	36.38 ± 6.04	0.735	0.08
SDS	39.03 ± 9.24	37.13 ± 8.45	0.340	0.21

Data are represented as Mean ± SD, *p < 0.05. MMSE, Mini-Mental State Exam; MoCA, Montreal Cognitive Assessment; AVLT, Auditory Verbal Learning Test; CFT, Complex Figure Test; DST, Digit Span Test. TMT-A, Trail Making Test-Part A; TMT-B, Trail Making Test-Part B; CDT, Clock Drawing Test; VFT, Verbal Fluency Test; DSST, Digit Symbol Substitution Test; SDS, Self-Rating Depression Scale; SAS, Self-Rating Anxiety Scale; ES, effect size.

### Resting-State Networks

**Figure 2:**
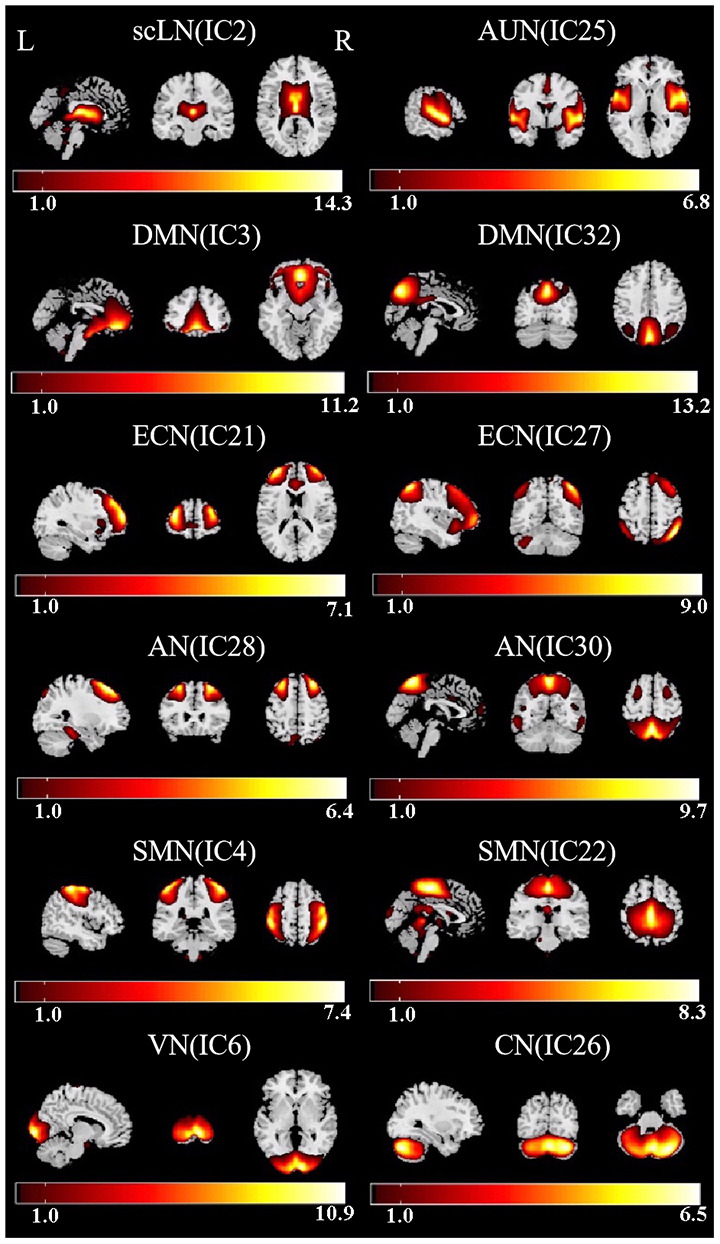
Functional relevant resting-state networks (RSNs). The spatial maps of 12 independent components (ICs) were selected as the RSNs for further analysis. AUN, Auditory network; DMN, default mode network; ECN, executive control network; AN, attention network; SMN, sensorimotor network; VN, visual network; CN, cerebellum network.

By using ICA approach, we obtained a cluster of 12 ICs and identified the eight meaningful RSNs (Figure 2), which is similar to previously reported research and including the following networks: the subcortical limbic network (scLN; IC2), which located throughout striatum and extends into the thalamus, brainstem, hippocampus, and amygdala. Auditory network (AUN; IC25) mainly consists of superior temporal gyrus and middle temporal gyrus which corresponded to the auditory system. The Default-mode network (DMN; IC3 + 32), includes the anterior region and the posterior region, the anterior region comprises the medial prefrontal cortex and the anterior cingulate cortex, and the posterior region mainly involves the posterior cingulate cortex/precuneus, bilateral inferior parietal cortex, and angular gyrus. The executive control network (ECN; IC21 + 27), primarily contains the bilateral dorsolateral prefrontal cortex and the lateral parietal cortex. Attention network (AN; IC28 + 30) is captured in two components, named as dorsal AN and ventral AN, including the following areas: the bilateral intraparietal sulcus, frontal eye field, ventral parietal cortex, and inferior frontal gyrus (IFG). Sensorimotor network (SMN; IC4 + 22) centered on the bilateral primary somatosensory cortex, mainly including precentral gyrus and postcentral gyrus. Also, visual network (VN; IC6) and cerebellum network (CN; IC26) were in agreement with the anatomical and functional delineations of the occipital lobe and cerebellar cortex, respectively.

### Altered FC Within RSNs

**Figure 3:**
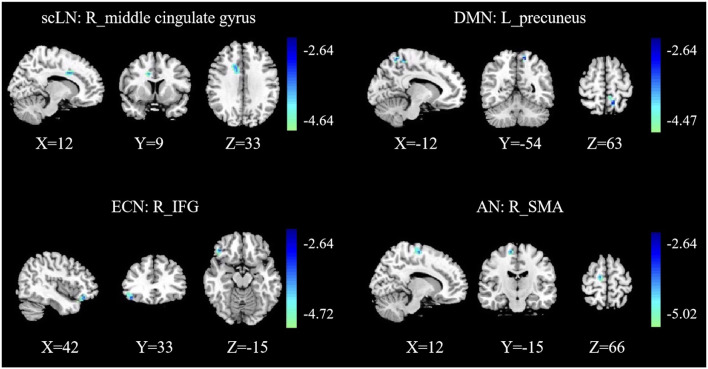
Group functional connectivity (FC) differences within RSNs. Significant differences between the presbycusis and control groups were found within four RSNs. scLN, subcortical limbic network; DMN, default mode network; ECN, executive control network; AN, attention network; IFG, inferior frontal gyrus; SMA, supplementary motor area; R, right; L, left.

Compared with the controls, presbycusis patients showed significant FC differences in four RSNs including the scLN, DMN, ECN, and AN, all of which revealed the decreased FC in the presbycusis group (Figure 3 and Table 3). For the scLN, FC decreased in the right middle cingulate gyrus (R_ middle cingulate gyrus). For the DMN, FC decreased in the left precuneus (L_Precuneus). For the ECN, FC decreased in the right Inferior Frontal Gyrus (R_IFG). And, the AN revealed the decreased FC in the right supplementary motor area (R_SMA). Besides, there were no differences in resting-state FC between controls and presbycusis groups within the AUN, SMN, VN, and CN.

**Table 3:** Brain regions with significant differences connectivity within resting-state networks (RSNs) between presbycusis patients and healthy controls.

	Brain regions	BA	Peak MNI coordinates			Peak T-value	Voxels
			*x*	*y*	*z*		
scLN	R_middle cingulate gyrus	32	12	9	33	−3.667	41
DMN	L_precuneus	7	−12	−54	63	−4.1241	41
ECN	R_IFG	47	42	33	−15	−3.8975	47
AN	R_SMA	6	12	−15	66	−4.0499	40

*MNI, Montreal Neurologic Institute; scLN, subcortical limbic network; DMN, default mode network; ECN, executive control network; AN, attention network; IFG, inferior frontal gyrus; SMA, supplementary motor area; R, right; L, left*.

### Altered Inter-network FC

**Figure 4:**
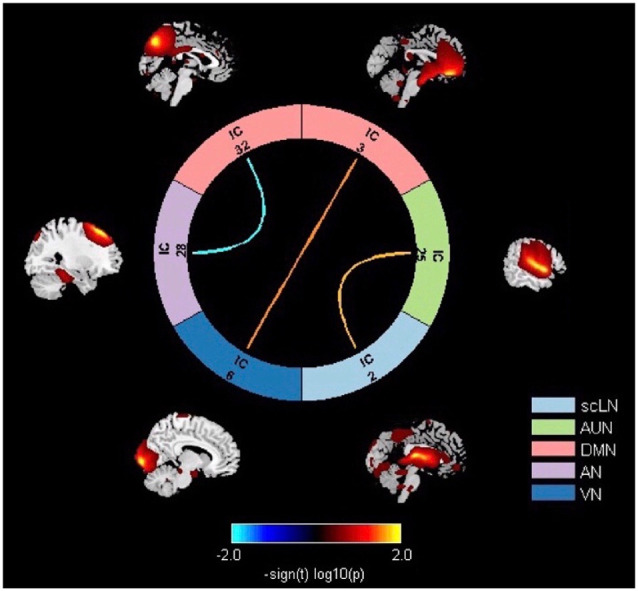
Significant differences in thenetwork connectivity in the subcortical limbic network (scLN), Auditory Network (AUN), default mode network (DMN), attention network (AN), and Visual Network (VN) between presbycusis and control groups.

Significant differences in the network connectivity in scLN, AUN, DMN, AN, and VN between presbycusis and control groups for FNC analysis were found (Figure 4). Subsequent analysis for significant differences in FNC revealed increased connectivity between scLN (IC2) and AUN (IC25), as well as the VN (IC6) and DMN (IC3) in presbycusis group compared to control group. Relative to the control group, the presbycusis group exhibited significantly decreased inter-network connectivity in the AN (28)-DMN (32).

### Correlation Analysis

Pearson or Spearman correlations were performed between the mean z-scores of 12 ROIs in the eight RSNs and cognitive assessment scores. Before multiple comparisons correction, significant correlations were found between the left precuneus within the DMN and DST scores (rho = 0.501, *p* = 0.001), as well as between the right IFG within the ECN and TMT-B scores (*r* = −0.341, *p* = 0.042). Moreover, after performing the correlations between the FNC coefficients (three connections) and the cognitive scores in presbycusis patients, only the AN-DMN connection was found to be positively correlated with DST scores (rho = 0.327, *p* = 0.040). Nevertheless, no significant correlations persisted after Bonferroni correction. Also, no significant associations between FD value and network connectivity in each RSNs were found in this study.

## Discussion

To our knowledge, this study is the first time using the ICA approach to detect the intra- and inter-network FC and their relationship with cognitive function in patients with presbycusis. Four brain networks were found to be abnormal in presbycusis patients compared with the controls, including scLN, DMN, ECN, and AN. Meanwhile, increased or decreased alterations of the inter-network functional coupling were found in the three functional connections in presbycusis patients through FNC analysis. And in presbycusis patients, only DST and TMT-B were found significant in cognitive performance, which may indicate that cognitive impairment is mainly manifested in the level of working memory and executive control function.

Analysis of RSNs internal FC focuses on the interaction of multiple networks in a certain spatial pattern (Beckmann et al., 2005). In this study, presbycusis patients observed reduced FC in the right middle cingulate gyrus for scLN. It is well known that scLN carries a variety of functions, including emotion, behavioral motivation, cognition, and memory processing (Morgane et al., 2005). A previous study found that in patients with sudden sensorineural hearing loss showed increased nodal betweenness of the limbic network, which may suggest a plastic compensatory mechanism to lessen the consequences of nerve damage caused by hearing loss and help maintain the patient’s cognitive abilities (Xu et al., 2016). Our research found reduced FC in the middle cingulate gyrus, suggesting the possibility of cognitive impairment in presbycusis patients. The middle cingulate gyrus forms an important component of the limbic system and is widely connected to neighboring brain regions *via* bundles of white matter fibers (Powell et al., 2018). Moreover, this study believes that the change stems from hearing impairment patients with persistent pressure on speech perception, which leads to reduced adaptive activity in the limbic network involved in the emotional processing of sounds.

As we know, the DMN controls both primary perception and advanced cognition and is responsible for the integration of these two (Zhao et al., 2018). Studies by Chen et al. (2018) indicated that compared with healthy controls, patients with hearing impairment showed reduced spontaneous neuronal activity in the precuneus. Also, decreased FC between the dorsal AN and the precuneus in patients with hearing loss has been emerging understanding. It is however not the hearing loss itself associated with reduced FC but the individually perceived listening effort that occurs as a result of hearing loss (Rosemann and Thiel, 2019). Moreover, the precuneus is functionally involved in episodic memory retrieval, self-awareness and processing, and is a critical element of the DMN (Cunningham et al., 2017; Feldstein Ewing and Chung, 2019). Meanwhile, we also found that the FC in the left precuneus within the DMN was associated with the DST score, implicating the disrupted working memory in presbycusis patients.

The ECN is involved in numerous advanced cognitive tasks and plays an important role in adaptive cognitive control (McHugh et al., 2017). In the current study, decreased FC in the right IFG within the ECN in patients with presbycusis was appeared, and significant correlations between the right IFG and TMT-B scores were found, reflecting the abnormality of the executive control function, which is inconsistent with the previous research. A psychiatric study (Rutherford et al., 2018) identified that hearing loss leads to reduced activation in central auditory pathways, thus resulting in compensatory increased activation in the ECN. Rosemann and Thiel (2019) observed that patients with hearing loss actively recruited frontal lobe regions, which possibly reflect increased efforts in executive function. Our results actually recognized a hypothesized mechanism for the relationship between presbycusis and cognitive impairment: the reallocation of cognitive resources. External acoustic stimuli reduced as a result of hearing loss, so more neural resources are allocated to deal with the attenuation of auditory signals, while few resources correspondingly left that can be used for higher-level cognition. The right IFG is mainly implicated in sensory input processing related to expectation and attention. The decline of its function indicates the impairment of attention and the impairment of language processing (Sherman et al., 2016; Briggs et al., 2019).

A growing body of evidence suggests that SMA is not only a motor structure but also involves in a wide range of cognitive fields (Bonini et al., 2014; Leek et al., 2016). It is generally believed that the processing of auditory is modulated by movement (Schneider and Mooney, 2018). A previous study discovered that the reduced directed FC from the SMA to the hippocampus revealed impaired sensorimotor function in patients with presbycusis (Chen et al., 2020). Panouillères and Mættænen suggested that in the old adults with hearing loss, the reduction of auditory input from the cochlea to the auditory system leads to a reduction in the recruitment of the articulatory cortex (Panouillères and Mættænen, 2018). This finding supports the hypothesis of auditory-motor decline (Skipper et al., 2017), which is in line with our results. The frontal regions depart from the primary motor cortex, passes through the intermediate premotor and SMA areas, and then head towards the prefrontal areas, thus to form an organizational structure (Fuster, 2006). Therefore, the decreased FC of SMA in presbycusis patients suggested the disruption of this organizational structure, leading to the impaired function of frontal lobe-mediated cognitive processing. The above results suggested that these four cognitive-related networks may have certain specificity in cognitive impairment mediated by presbycusis, which will help understand the neuropathological mechanism of presbycusis.

Recently, gradually increasing evidence suggested that RSNs are interdependent and continue to interact with each other (Smith et al., 2015). Our results illustrated the increased connectivity for the scLN-AUN, which may be following the conceptual model proposed by Jastreboff (1990). Our results revealed that under the condition of long-term deprivation of auditory ability and insufficient auditory input in presbycusis patients, the compensatory feedback neural activity of the scLN increased, reflecting a certain degree of plastic reorganization within the limbic network. Data from previous animal models also indicated that hearing loss promotes cortical reorganization and morphological alterations (Peelle and Wingfield, 2016). Besides, it also suggested that the auditory cortex is involved in the analysis of inputs from higher-order functions mediated by scLN. Besides, our study also demonstrated the hyper-connectivity for VN-DMN, reflecting the improvement of visual motion or peripheral spatial localization ability of patients with presbycusis, and the compensatory plastic reorganization occurs in the brain network which may be due to the lack of auditory ability. The strengthened functional coupling of the local sensory network including visual and sensory-motor areas in patients with sloping sensorineural hearing loss indicates that the auditory-deprived brain will have integrated compensation for the remaining sensory regions (Wolak et al., 2019).

The decline in attention and the reduction in the volume of the attention-related cortex reflects the impaired function of the AN (Cardin, 2016), which in turn affects the perceptual analysis and the auditory processing of acoustic signals, always occurs in presbycusis patients (Fortunato et al., 2016). Previously investigating the cross-network interaction between the DMN and the DAN, it was found that activities in DMN decreased when performing goal-directed tasks, while activities in DAN increased. This relationship suggested to be anti-correlated (Fox et al., 2009), and our result seems to reinforce this decoupling effect. That is, the FC in AN reduced to stabilize the balance from the external environment, such as auditory input. However, the reduction of perceived auditory tasks in presbycusis leads to the interruption of AN, and decrease the connectivity to key nodes of the DMN. The interruption of AN is also one of the causes of memory impairment (Veldsman et al., 2019). In addition to FNC abnormalities, a negative correlation between the AN-DMN and DST scores was also detected, showing that the dis-coupling probably relates to the disrupted working memory in presbycusis patients, and these disconnections above may also contribute to daily cognitive impairment.

Several limitations should be paid attention to. First of all, although the FC results are consistent with the previous literature, the essence of the interaction between and within different internal connectivity networks has poorly comprehended. Second, the brain networks involved in this study are limited. Because other networks may play an important role in the pathophysiology of cognitive impairment in the presbycusis, detecting the variation of the FC in multiple networks will provide insight into the neural mechanism in depth. Further researches should focus on the effects of presbycusis on other brain networks and conduct longitudinal research to predict long-term cognitive function. Moreover, no significant results persisted after Bonferroni correction for multiple comparisons in the correlation analyses due to the relatively strict method, which will be considered in further study. Nonetheless, our research is still meaningful to provide some enlightenment for future studies in this field. Finally, the newly reported rs-FC is difficult to reflect the characteristics of temporal variations within the brain network, therefore, dynamic FNC is regarded as the future research direction. Also, the directionality of the interaction between networks cannot reveal by the ICA approach, further studies are needed to assess the specific and directional function in the coupling between brain networks.

To sum up, this study suggested that the structure of intra- and inter-networks FC of presbycusis patients has undergone profound alterations. The alterations involving the primarily auditory system and other high-order cognitive control networks have demonstrated that brain network with extensive plastic reorganization during the abnormal state, which provides meaningful insights for further understanding the neural mechanism of cognitive impairment in presbycusis patients.

## Data Availability Statement

The raw data supporting the conclusions of this article will be made available by the authors, without undue reservation.

## Ethics Statement

The studies involving human participants were reviewed and approved by the local institutional review board of Nanjing Medical University. The patients/participants provided their written informed consent to participate in this study. Written informed consent was obtained from the individual(s) for the publication of any potentially identifiable images or data included in this article.

## Author Contributions

CX and JZ designed the experiment, collected the data, performed the analysis and wrote the manuscript. JC, WY, JH, and XY collected the data. YW and Y-CC contributed to the discussion and manuscript revision.

## Conflict of Interest

The authors declare that the research was conducted in the absence of any commercial or financial relationships that could be construed as a potential conflict of interest.

## Footnotes

^1^http://icatb.sourceforge.net

^2^https://afni.nimh.nih.gov/pub/dist/doc/manual/AlphaSim.pdf

## References

[B1] Ardila A. Bernal B. Rosselli M. (2016). How localized are language brain areas? A review of brodmann areas involvement in oral language. Arch. Clin. Neuropsychol. 31, 112–122. 10.1093/arclin/acv081 26663825

[B2] Beckmann C. F. DeLuca M. Devlin J. T. Smith S. M. (2005). Investigations into resting-state connectivity using independent component analysis. Philos. Trans. R. Soc. Lond. B Biol. Sci. 360, 1001–1013. 10.1098/rstb.2005.1634 16087444 PMC1854918

[B3] Bidelman G. M. Mahmud M. S. Yeasin M. Shen D. Arnott S. R. Alain C. (2019). Age-related hearing loss increases full-brain connectivity while reversing directed signaling within the dorsal-ventral pathway for speech. Brain Struct. Funct. 224, 2661–2676. 10.1007/s00429-019-01922-9 31346715 PMC6778722

[B4] Biswal B. Yetkin F. Z. Haughton V. M. Hyde J. S. (1995). Functional connectivity in the motor cortex of resting human brain using echo-planar MRI. Magn. Reson. Med. 34, 537–541. 10.1002/mrm.1910340409 8524021

[B5] Bonini F. Burle B. Liégeois-Chauvel C. Régis J. Chauvel P. Vidal F. (2014). Action monitoring and medial frontal cortex: leading role of supplementary motor area. Science 343, 888–891. 10.1126/science.1247412 24558161

[B6] Briggs R. G. Chakraborty A. R. Anderson C. D. Abraham C. J. Palejwala A. H. Conner A. K.. (2019). Anatomy and white matter connections of the inferior frontal gyrus. Clin. Anat. 32, 546–556. 10.1002/ca.23349 30719769

[B7] Brucki S. M. Rocha M. S. (2004). Category fluency test: effects of age, gender and education on total scores, clustering and switching in Brazilian Portuguese-speaking subjects. Braz. J. Med. Biol. Res. 37, 1771–1777. 10.1590/s0100-879x2004001200002 15558183

[B8] Cardin V. (2016). Effects of aging and adult-onset hearing loss on cortical auditory regions. Front. Neurosci. 10:199. 10.3389/fnins.2016.00199 27242405 PMC4862970

[B9] Chen Y. C. Chen H. Jiang L. Bo F. Xu J. J. Mao C. N.. (2018). Presbycusis disrupts spontaneous activity revealed by resting-state functional MRI. Front. Behav. Neurosci. 12:44. 10.3389/fnbeh.2018.00044 29593512 PMC5859072

[B10] Chen Y. C. Yong W. Xing C. Feng Y. Haidari N. A. Xu J. J.. (2020). Directed functional connectivity of the hippocampus in patients with presbycusis. Brain Imaging Behav. 14, 917–926. 10.1007/s11682-019-00162-z 31270776

[B11] Cunningham S. I. Tomasi D. Volkow N. D. (2017). Structural and functional connectivity of the precuneus and thalamus to the default mode network. Hum. Brain Mapp. 38, 938–956. 10.1002/hbm.23429 27739612 PMC6866740

[B13] Feldstein Ewing S. W. Chung T. (2019). Precuneus: a key on the road to translation. Alcohol. Clin. Exp. Res. 43, 1063–1065. 10.1111/acer.14026 30892706 PMC6684171

[B14] Ford A. H. Hankey G. J. Yeap B. B. Golledge J. Flicker L. Almeida O. P. (2018). Hearing loss and the risk of dementia in later life. Maturitas 112, 1–11. 10.1016/j.maturitas.2018.03.004 29704910

[B15] Fortunato S. Forli F. Guglielmi V. De Corso E. Paludetti G. Berrettini S.. (2016). A review of new insights on the association between hearing loss and cognitive decline in ageing. Acta Otorhinolaryngol. Ital. 36, 155–166. 10.14639/0392-100X-993 27214827 PMC4977003

[B16] Fox M. D. Zhang D. Snyder A. Z. Raichle M. E. (2009). The global signal and observed anticorrelated resting state brain networks. J. Neurophysiol. 101, 3270–3283. 10.1152/jn.90777.2008 19339462 PMC2694109

[B17] Fuster J. M. (2006). The cognit: a network model of cortical representation. Int. J. Psychophysiol. 60, 125–132. 10.1016/j.ijpsycho.2005.12.015 16626831

[B18] Gabel N. M. Waldron-Perrine B. Spencer R. J. Pangilinan P. H. Hale A. C. Bieliauskas L. A. (2019). Suspiciously slow: timed digit span as an embedded performance validity measure in a sample of veterans with mTBI. Brain Inj. 33, 377–382. 10.1080/02699052.2018.1553311 30526120

[B19] Gates G. A. Mills J. H. (2005). Presbycusis. Lancet 366, 1111–1120. 10.1016/S0140-6736(05)67423-5 16182900

[B12] GBD 2015 Disease and Injury Incidence and Prevalence Collaborators. (2016). Global, regional, and national incidence, prevalence and years lived with disability for 310 diseases and injuries, 1990–2015: a systematic analysis for the Global Burden of Disease Study 2015. Lancet 388, 1545–1602. 10.1016/S0140-6736(16)31678-6 27733282 PMC5055577

[B20] Geerligs L. Renken R. J. Saliasi E. Maurits N. M. Lorist M. M. (2015). A brain-wide study of age-related changes in functional connectivity. Cereb. Cortex 25, 1987–1999. 10.1093/cercor/bhu012 24532319

[B21] Jastreboff P. J. (1990). Phantom auditory perception (tinnitus): mechanisms of generation and perception. Neurosci. Res. 8, 221–254. 10.1016/0168-0102(90)90031-9 2175858

[B22] Leek E. C. Yuen K. S. Johnston S. J. (2016). Domain general sequence operations contribute to pre-SMA involvement in visuo-spatial processing. Front. Hum. Neurosci. 10:9. 10.3389/fnhum.2016.00009 26858623 PMC4727040

[B23] Lopez-Escamez J. A. Carey J. Chung W. H. Goebel J. A. Magnusson M. Mandala M.. (2015). Diagnostic criteria for Meniere’s disease. J. Vestib. Res. 25, 1–7. 10.3233/VES-150549 25882471

[B24] Loughrey D. G. Kelly M. E. Kelley G. A. Brennan S. Lawlor B. A. (2018). Association of age-related hearing loss with cognitive function, cognitive impairment and dementia: a systematic review and meta-analysis. JAMA Otolaryngol. Head Neck Surg. 144, 115–126. 10.1001/jamaoto.2017.2513 29222544 PMC5824986

[B25] Lu J. Li D. Li F. Zhou A. Wang F. Zuo X.. (2011). Montreal cognitive assessment in detecting cognitive impairment in Chinese elderly individuals: a population-based study. J. Geriatr. Psychiatry Neurol. 24, 184–190. 10.1177/0891988711422528 22228824

[B26] Luan Y. Wang C. Jiao Y. Tang T. Zhang J. Teng G. J. (2019). Dysconnectivity of multiple resting-state networks associated with higher-order functions in sensorineural hearing loss. Front. Neurosci. 13:55. 10.3389/fnins.2019.00055 30804740 PMC6370743

[B27] Lv H. Wang Z. Tong E. Williams L. M. Zaharchuk G. Zeineh M.. (2018). Resting-state functional MRI: everything that nonexperts have always wanted to know. Am. J. Neuroradiol. 39, 1390–1399. 10.3174/ajnr.a5527 29348136 PMC6051935

[B28] McHugh M. J. Gu H. Yang Y. Adinoff B. Stein E. A. (2017). Executive control network connectivity strength protects against relapse to cocaine use. Addict. Biol. 22, 1790–1801. 10.1111/adb.12448 27600492

[B29] McKeown M. J. Makeig S. Brown G. G. Jung T. P. Kindermann S. S. Bell A. J.. (1998). Analysis of fMRI data by blind separation into independent spatial components. Hum. Brain Mapp. 6, 160–188. 10.1002/(SICI)1097-0193(1998)6:3<160::AID-HBM5>3.0.CO;2-1 9673671 PMC6873377

[B30] Morgane P. J. Galler J. R. Mokler D. J. (2005). A review of systems and networks of the limbic forebrain/limbic midbrain. Prog. Neurobiol. 75, 143–160. 10.1016/j.pneurobio.2005.01.001 15784304

[B31] Panouillères M. T. N. Mættænen R. (2018). Decline of auditory-motor speech processing in older adults with hearing loss. Neurobiol. Aging 72, 89–97. 10.1016/j.neurobiolaging.2018.07.013 30240945

[B32] Peelle J. E. Wingfield A. (2016). The neural consequences of age-related hearing loss. Trends Neurosci. 39, 486–497. 10.1016/j.tins.2016.05.001 27262177 PMC4930712

[B33] Powell R. Elwes R. Hamandi K. Mullatti N. (2018). Cingulate gyrus epilepsy. Pract. Neurol. 18, 447–454. 10.1136/practneurol-2017-001812 30100562

[B34] Qin Y. Li Y. Sun B. He H. Peng R. Zhang T.. (2018). Functional connectivity alterations in children with spastic and dyskinetic cerebral palsy. Neural Plast. 2018:7058953. 10.1155/2018/7058953 30186320 PMC6114065

[B35] Rosano C. Chang Y. F. Kuller L. H. Guralnik J. M. Studenski S. A. Aizenstein H. J.. (2013). Long-term survival in adults 65 years and older with white matter hyperintensity: association with performance on the digit symbol substitution test. Psychosom. Med. 75, 624–631. 10.1097/psy.0b013e31829c1df2 23886735 PMC3809761

[B36] Rosemann S. Thiel C. M. (2019). The effect of age-related hearing loss and listening effort on resting state connectivity. Sci. Rep. 9:2337. 10.1038/s41598-019-38816-z 30787339 PMC6382886

[B37] Rutherford B. R. Brewster K. Golub J. S. Kim A. H. Roose S. P. (2018). Sensation and psychiatry: linking age-related hearing loss to late-life depression and cognitive decline. Am. J. Psychiatry 175, 215–224. 10.1176/appi.ajp.2017.17040423 29202654 PMC5849471

[B38] Sánchez-Cubillo I. Periáñez J. A. Adrover-Roig D. Rodríguez-Sánchez J. M. Ríos-Lago M. Tirapu J.. (2009). Construct validity of the Trail Making Test: role of task-switching, working memory, inhibition/interference control, and visuomotor abilities. J. Int. Neuropsychol. Soc. 15, 438–450. 10.1017/s1355617709090626 19402930

[B39] Schmidt S. A. Akrofi K. Carpenter-Thompson J. R. Husain F. T. (2013). Default mode, dorsal attention and auditory resting state networks exhibit differential functional connectivity in tinnitus and hearing loss. PLoS One 8:e76488. 10.1371/journal.pone.0076488 24098513 PMC3788711

[B40] Schneider D. M. Mooney R. (2018). How movement modulates hearing. Annu. Rev. Neurosci. 41, 553–572. 10.1146/annurev-neuro-072116-031215 29986164 PMC6201761

[B41] Sherman M. T. Seth A. K. Kanai R. (2016). Predictions shape confidence in right inferior frontal gyrus. J. Neurosci. 36, 10323–10336. 10.1523/JNEUROSCI.1092-16.2016 27707969 PMC6705584

[B42] Shin M. S. Park S. Y. Park S. R. Seol S. H. Kwon J. S. (2006). Clinical and empirical applications of the rey-osterrieth complex figure test. Nat. Protoc. 1, 892–899. 10.1038/nprot.2006.115 17406322

[B43] Skipper J. I. Devlin J. T. Lametti D. R. (2017). The hearing ear is always found close to the speaking tongue: review of the role of the motor system in speech perception. Brain Lang. 164, 77–105. 10.1016/j.bandl.2016.10.004 27821280

[B44] Smith S. M. Nichols T. E. Vidaurre D. Winkler A. M. Behrens T. E. Glasser M. F.. (2015). A positive-negative mode of population covariation links brain connectivity, demographics and behavior. Nat. Neurosci. 18, 1565–1567. 10.1038/nn.4125 26414616 PMC4625579

[B45] SongH. J.MeadeK.AkobunduU.SahyounN. R. (2014). Depression as a correlate of functional status of community-dwelling older adults: utilizing a short-version of 5-item Geriatric Depression Scale as a screening tool. J. Nutr. Health Aging 18, 765–770. 10.1007/s12603-014-0542-025286457

[B46] Tavanai E. Mohammadkhani G. (2017). Role of antioxidants in prevention of age-related hearing loss: a review of literature. Eur. Arch. Otorhinolaryngol. 274, 1821–1834. 10.1007/s00405-016-4378-6 27858145

[B47] Thomson R. S. Auduong P. Miller A. T. Gurgel R. K. (2017). Hearing loss as a risk factor for dementia: a systematic review. Laryngoscope Investig. Otolaryngol. 2, 69–79. 10.1002/lio2.65 28894825 PMC5527366

[B48] Veldsman M. Zamboni G. Butler C. Ahmed S. (2019). Attention network dysfunction underlies memory impairment in posterior cortical atrophy. Neuroimage Clin. 22:101773. 10.1016/j.nicl.2019.101773 30991615 PMC6453667

[B49] ViscogliosiG.Di BernardoM. G.EttorreE.ChiriacI. M. (2017). Handgrip strength predicts longitudinal changes in clock drawing test performance. An observational study in a sample of older non-demented adults. J. Nutr. Health Aging 21, 593–596. 10.1007/s12603-016-0816-928448092

[B50] Wang C. Qin W. Zhang J. Tian T. Li Y. Meng L.. (2014). Altered functional organization within and between resting-state networks in chronic subcortical infarction. J. Cereb. Blood Flow Metab. 34, 597–605. 10.1038/jcbfm.2013.238 24398939 PMC3982082

[B51] Wang J. Wang X. Xia M. Liao X. Evans A. He Y. (2015). GRETNA: a graph theoretical network analysis toolbox for imaging connectomics. Front. Hum. Neurosci. 9:386. 10.3389/fnhum.2015.00386 26175682 PMC4485071

[B52] Wolak T. Cieśla K. Pluta A. Włodarczyk E. Biswal B. Skarzyński H. (2019). Altered functional connectivity in patients with sloping sensorineural hearing loss. Front. Hum. Neurosci. 13:284. 10.3389/fnhum.2019.00284 31507391 PMC6713935

[B54] Xu Y. Chen K. Zhao Q. Li F. Guo Q. (2020). Short-term delayed recall of auditory verbal learning test provides equivalent value to long-term delayed recall in predicting MCI clinical outcomes: a longitudinal follow-up study. Appl. Neuropsychol. Adult 27, 73–81. 10.1080/23279095.2018.1481067 30470140

[B53] Xu H. Fan W. Zhao X. Li J. Zhang W. Lei P.. (2016). Disrupted functional brain connectome in unilateral sudden sensorineural hearing loss. Hear. Res. 335, 138–148. 10.1016/j.heares.2016.02.016 26969260

[B55] Zhao Z. Wu J. Fan M. Yin D. Tang C. Gong J.. (2018). Altered intra- and inter-network functional coupling of resting-state networks associated with motor dysfunction in stroke. Hum. Brain Mapp. 39, 3388–3397. 10.1002/hbm.24183 29691945 PMC6866540

[B56] Zung W. W. (1971). A rating instrument for anxiety disorders. Psychosomatics 12, 371–379. 10.1016/s0033-3182(71)71479-0 5172928

